# Infections and Autoimmunity—The Immune System and Vitamin D: A Systematic Review

**DOI:** 10.3390/nu15173842

**Published:** 2023-09-02

**Authors:** Sunil J. Wimalawansa

**Affiliations:** Medicine, Endocrinology & Nutrition, Cardiometabolic & Endocrine Institute, North Brunswick, NJ 08902, USA; suniljw@hotmail.com or sunil.wimalawansa@rutgers.edu

**Keywords:** 25(OH)D, epidemiology, morbidity, mortality, prevention, treatment, public health

## Abstract

Both 25-autoimmunity and(25(OH)D: calcifediol) and its active form, 1,25-dihydroxyvitamin D (1,25(OH)_2_D: calcitriol), play critical roles in protecting humans from invasive pathogens, reducing risks of autoimmunity, and maintaining health. Conversely, low 25(OH)D status increases susceptibility to infections and developing autoimmunity. This systematic review examines vitamin D’s mechanisms and effects on enhancing innate and acquired immunity against microbes and preventing autoimmunity. The study evaluated the quality of evidence regarding biology, physiology, and aspects of human health on vitamin D related to infections and autoimmunity in peer-reviewed journal articles published in English. The search and analyses followed PRISMA guidelines. Data strongly suggested that maintaining serum 25(OH)D concentrations of more than 50 ng/mL is associated with significant risk reduction from viral and bacterial infections, sepsis, and autoimmunity. Most adequately powered, well-designed, randomized controlled trials with sufficient duration supported substantial benefits of vitamin D. Virtually all studies that failed to conclude benefits or were ambiguous had major study design errors. Treatment of vitamin D deficiency costs less than 0.01% of the cost of investigation of worsening comorbidities associated with hypovitaminosis D. Despite cost-benefits, the prevalence of vitamin D deficiency remains high worldwide. This was clear among those who died from COVID-19 in 2020/21—most had severe vitamin D deficiency. Yet, the lack of direction from health agencies and insurance companies on using vitamin D as an adjunct therapy is astonishing. Data confirmed that keeping an individual’s serum 25(OH)D concentrations above 50 ng/mL (125 nmol/L) (and above 40 ng/mL in the population) reduces risks from community outbreaks, sepsis, and autoimmune disorders. Maintaining such concentrations in 97.5% of people is achievable through daily safe sun exposure (except in countries far from the equator during winter) or taking between 5000 and 8000 IU vitamin D supplements daily (average dose, for non-obese adults, ~70 to 90 IU/kg body weight). Those with gastrointestinal malabsorption, obesity, or on medications that increase the catabolism of vitamin D and a few other specific disorders require much higher intake. This systematic review evaluates non-classical actions of vitamin D, with particular emphasis on infection and autoimmunity related to the immune system.

## 1. Introduction 

In humans, most of the vitamin D requirement is expected to be generated by summer-like sunlight, with at least a third of the upper body exposed to direct sunlight [1]. The best time for sun exposure is between 10.30 a.m. and 1.30 p.m. (when one’s shadow is shorter than the height), when the sun’s ultraviolet B rays (UVB) come at a narrow (zenith) angle, allowing better skin penetration [2,3]. Since most vitamin D should be derived from UVB rays from the sun, insufficient exposure is the most typical cause of vitamin D deficiency [4,5,6,7]. Nevertheless, most people have inherent sun avoidance behavior, making it worse. While 40% of citizens in Western countries take supplements (mostly insufficient doses), less than 5% do so in developing countries. Over 50% of the world’s population has suboptimal vitamin D concentrations at a given time [8,9]. 

Therefore, globally, vitamin D deficiency is a significant public health problem—a pandemic—that has overtaken iron deficiency as the most common nutritional deficiency in the world. As described below, vitamin D deficiency is associated with many chronic diseases and significantly increases the risk of infections [10,11,12,13]. Despite these, no government or medical insurance companies address this vital public health issue—hypovitaminosis D. It increases chronic ill-health, absenteeism, and healthcare costs. Nevertheless, with proper public health guidance related to safe sun exposure and supplements, vitamin D deficiency can be eliminated cost-effectively, thus reducing morbidities, premature deaths, and healthcare costs. 

25(OH)D is further hydroxylated into a multifunctional seco-steroidal hormone in renal tubular cells. This is essential for musculoskeletal and parathyroid functions [14,15,16,17]. The prevalence of vitamin D deficiency and its associated complications have been escalating over the past three decades and affecting globally. This study was undertaken in part to address this issue. This systematic review focuses on infections and autoimmune disorders related to hypovitaminosis D. 

### 1.1. Systematic Review Process

PubMed, Medline, Google Scholar, and EMBASE databases were searched for randomized controlled clinical trials (RTCs), prospective clinical studies, and original and review articles related to vitamin D, infections, autoimmunity, and the immune system. The study was conducted as per the Preferred Reporting Items for Systematic Reviews and Meta-Analyses (PRISMA) statement [18,19], the Equator Network (www.equator-network.org/ (accessed on 5 March 2023), the PRISMA statement [20], and the PRISMA-P guidelines and checklist [18,21]. Search terms used include the controlled words vitamin D, cholecalciferol, 25(OH)D, 25-hydroxyvitamin D, 25-hydroxycholecalciferol, calcitriol, calcifediol, and calcidiol, in conjunction with infections, autoimmunity, and the immune system. These were selected from the EMTREE thesaurus, Medical Subject Headings terms [22]. Keywords were used in combinations of two to reduce the number of manuscripts to a workable number. 

### 1.2. Protocol and Manuscript Selection

**Figure 1:**
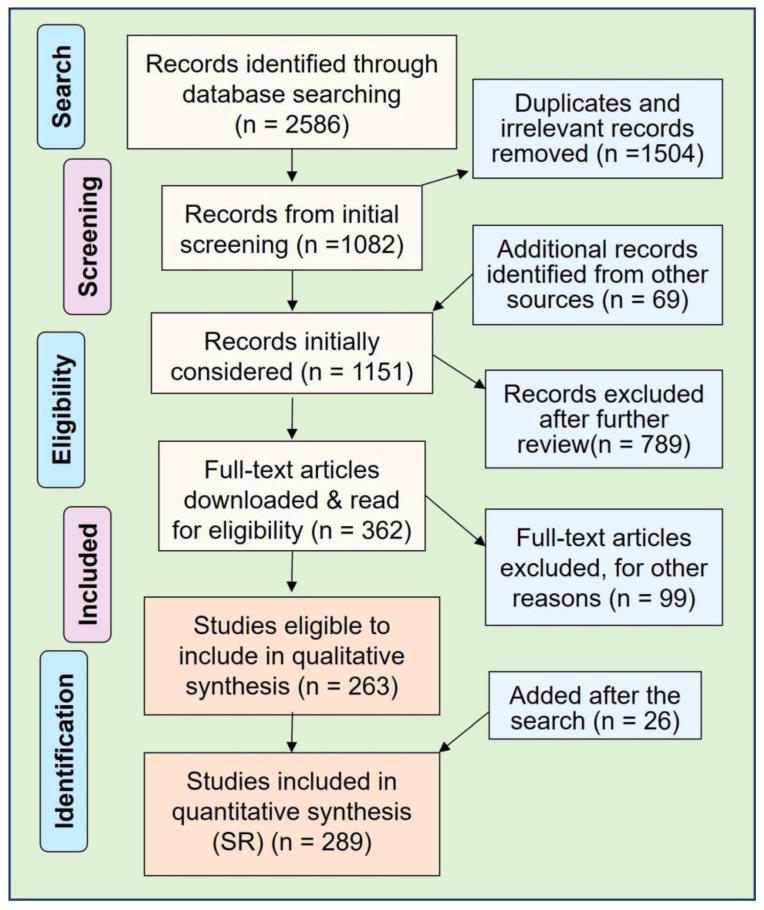
PRISMA flow chart. Selection path of reference to advances in knowledge of vitamin D with particular emphasis on infections, autoimmunity, and the immune system.

A protocol was developed to track pertinent areas and publications. The manuscript selection included groups covering RCTs, observational, ecological, and epidemiological studies, and supporting laboratory and animal studies [19]. Following the literature search, the quality and relevance of studies on the topic were assessed, and a catalog was developed with manuscripts [18]. Articles published between 1 January 2000 and May 2023 in English were searched. After removing duplicates, the screening produced 2586 manuscripts from combined databases. The removal of 1504 and further 789 later records due to duplicates and lack of direct relevance led to 263 qualified articles for the SR—an additional 26 articles were included after the search till the end of July 2023. Two hundred eighty-nine articles were included in the final manuscript using EndNote 20.6 reference manager programs (Figure 1). 

### 1.3. Search, Data Abstraction, Synthesis, and Scope

Rationale, eligibility, and exclusion criteria for the evidence-based PICO process (patient problems, intervention, comparison or control, and outcome elements) were observed, and potential bias in individual studies and design failures were noted for exclusion [18,21]. The strength of the evidence concerning the biology and physiology of vitamin D related to human health, specifically immunity, autoimmunity, and combatting infections, was assessed. Synthesized results were included as narrative conclusions [18]. 

In addition to the association between vitamin D and the musculoskeletal system, evidence is growing regarding the broader benefits of vitamin D. However, recently published larger RCTs, many with poor study designs, have muddled the field of vitamin D, referencing relationships and causation. This is mainly attributable to the study design failures [23,24,25,26,27] and some bias. PubMed searches with keywords and analysis revealed that the publications over the past 15 years related to non-classic vitamin D actions had far exceeded the classical action in the musculoskeletal system. In addition, nine out of ten non-classic vitamin D studies reported positive outcomes. 

**Figure 2:**
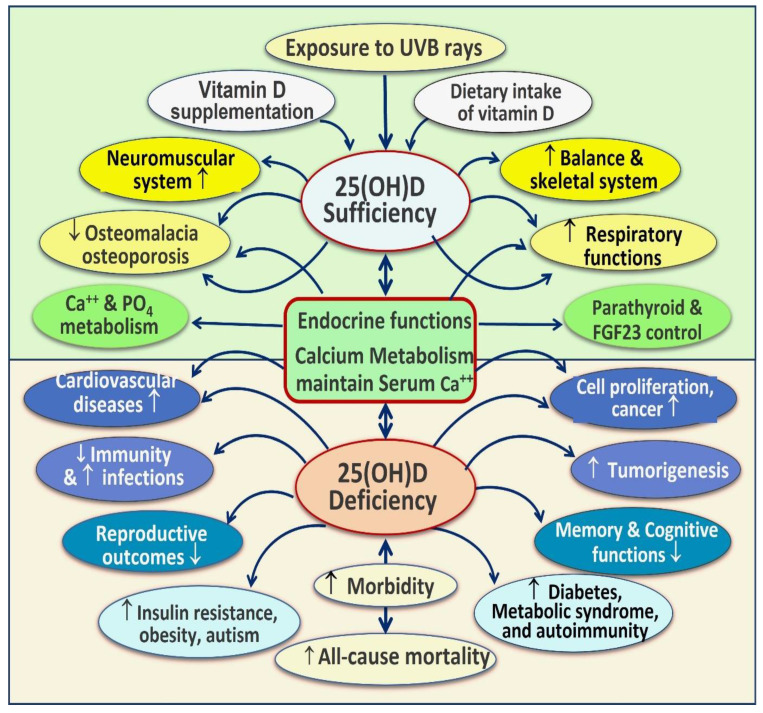
Relationships between vitamin D and a spectrum of non-skeletal diseases and disorders associated with vitamin D deficiency. The complicated relationships between beneficial 25(OH)D concentrations (sufficiency) and various organ systems in the body and diseases are depicted. Top panel (light green background)—vitamin D sufficiency: White ovals—mode of vitamin D generation/entry to the body. Yellow ovals—system dysfunction. Green ovals—endocrine functions of vitamin D (circulating 1,25(OH)_2_D: calcitriol) on calcium metabolism. Bottom panela (light yellow background)— vitamin D deficiency: Dark blue ovals—functional and pathophysiological relationships with tissues and organ systems. Light blue ovals—metabolic dysfunctions associated with hypovitaminosis D. Abbreviations: Ca^++^, calcium; FGF23, fibroblast growth factor-23; IR, insulin resistance; Mg^++^, magnesium; UV, ultraviolet rays. Arrows indicate increased (improved) or decreased incidence or severity (modified from Wimalawansa 2012 and 2016 [30,31]).

This study confirmed a strong association between vitamin D status (deficiency) with the initiation of autoimmunity and failures to combat infections, particularly viral and intracellular bacterial infections. The study also identified (a) the importance of higher-quality RCTs with proper clinical study designs to test hypotheses regarding health outcomes attributable to the nutrient vitamin D [1] and (b) the need for eliminating poorly designed studies from meta-analyses. Positive vs. negative outcomes were predictable based on the quality or errors of study designs. Whereas almost all well-designed, statistically powered RCTs provided anticipated positive clinical outcomes [28,29]. Figure 2 illustrates the body systems dependent on vitamin D sufficiency for proper functioning. Common disorders are worsened by chronic vitamin D deficiency. 

## 2. Vitamin D—Innate and Acquired Immunity

1,25-dihydroxycholecalciferol (calciferol) is the most active vitamin D metabolite and a potent immune modulator essential for combating pathogens [32,33]. As described below, the circulating hormonal form of calcitriol does not affect immune cell functions. The functionality of these cells depends on adequate generation of calcitriol within them. Calcitriol (a) activates cytosol’s vitamin D (calcitriol)receptors (VDRs) following translocation into the nucleus to modulate genomic functions, and (b) acts as signaling molecules for its non-genomic actions, like membrane effects, and autocrine and paracrine signaling.

Calcitriol concentrations in the circulation are controlled mainly by parathyroid hormone (PTH) partly via circulatory ionized calcium but not by tissue 24-hydroxylase. In contrast, in the target tissues, the production of calcitriol is mainly regulated by a combination of the circulatory 25(OH)D concentration (and vitamin D) and the feedback catabolic activity of tissue 24-hydroxylase enzyme and not by PTH or serum calcium. Vitamin D has no known action and, thus, is not measured routinely. Consequently, vitamin D physiologic correlations are focused on the serum 25(OH)D concentrations—“physiological concentrations of vitamin D status”.

### 2.1. Vitamin D Activates Immune Cells 

The interaction of calcitriol with its receptor leads to the translocation of the receptor complex to the nucleus, where it binds to the genome and modulates over 1200 genes [34]. Calcitriol down-regulates inflammation and oxidative stress through multiple mechanisms, primarily by suppressing inflammatory cytokines. The immunomodulatory effects of vitamin D include activation of immune cells such as T and B cells and macrophage and dendritic cells, as well as increased production of antimicrobial peptides and neutralizing antibodies [35,36,37]. 

As with certain vaccinations, like repeated bivalent COVID-19 booster doses, chronic hypovitaminosis D also causes immune paresis, increasing the vulnerability to infections, especially to intracellular bacteria such as tuberculosis and [38] and respiratory viruses [39,40], including SARS-CoV-2 [41,42]. Recent clinical studies have supported the latter [43]. The vulnerability to SARS-CoV-2 was reported in those who were PCR-positive, symptomatic SARS-CoV-2 infection, and had severe complications. They had a significantly higher prevalence of vitamin D deficiency—low serum 25(OH)D concentrations—mean concentration of 11.1 ng/mL; *p* = 0.004, compared with those with negative results (24.6 ng/mL) [44]. 

In addition, vulnerable people, such as older people with comorbidities, strongly correlate with low vitamin D status and cytokine storm—a hyper-inflammatory condition caused by an uncontrolled, overactive immune status [45]. Symptomatic disease, complications, and deaths from viral infections, including SARS-CoV-2, are based on the underlying vulnerability (i.e., weak immune system) and the viral load. Thus, while vitamin D does not prevent viral infections or a person from contracting COVID-19, it significantly reduces symptomatic disease, complications, and deaths [43,46,47,48,49,50,51]. 

It was reported that, in the period before the infection (e.g., immediate pre-pandemic), hypovitaminosis D increases these risks and vulnerability [52,53,54,55]. Besides, vitamin D deficiency at the time of diagnosis of SARS-CoV-2 infection significantly increased the severity and mortality [43,48,56,57]. In contrast, vitamin D sufficiency is protective against severe COVID-19 disease and deaths [46,48,58,59]. These data are relevant for establishing the Bradford Hill criteria [60]: vitamin D deficiency as a cause for infection, severity, and mortality from SARS-CoV-2 virus [43,46,47,48,49,50,51].

**Figure 3:**
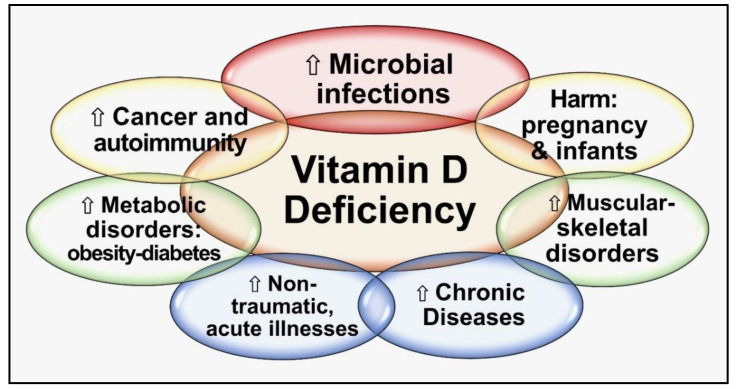
Major negative consequences are categorized into groups of chronic vitamin D deficiency.

Vitamin D controls autoimmunity by suppressing adaptive immunity via T- and B-lymphocyte activity [61]. Consequently, hypovitaminosis D leads to a dysfunctional immune system; the prime reason for initiating autoimmune responses [62,63,64,65,66]. In addition, having low serum 25(OH)D concentrations worsens existing autoimmune diseases [67,68], such as multiple sclerosis (MS) [69]. Hypovitaminosis D also increases risks for autoimmune diseases [65,66]. Persons with several autoimmune disorders, such as type 1 diabetes, autoimmune adrenal disease, MS, Hashimoto’s thyroiditis, etc., are known to have lower concentrations of serum 25(OH)D (calcifediol) [63,64,70]. These data strongly support an inverse relationship between vitamin D status and autoimmunity: the lower the serum 25(OH)D concentrations, the higher the risks for autoimmunity—both incidence and severity [64,65,66]. Figure 3 summarizes the critical negative outcomes of chronic vitamin D deficiency.

In contrast, vitamin D sufficiency reduces not only acute infections like SARS-CoV-2 but also the risks of chronic infections, such as tuberculosis. Sufficient generation of calcitriol within the immune cells regulates innate and adaptive immunity, potentiating the innate response (monocytes/macrophages with anti-microbial activity) [10,11,12,13,71]. Calcitriol also modulates B lymphocytes and plasma cells for immunoglobulin production and stabilizes B-cells [61,72], increasing anti-microbial peptide synthesis (Section 3.5 and Section 3.6).

### 2.2. Modes of Stimulating the Immune System by Vitamin D

Following the interactions of vitamin D and VDR and activating second messenger signaling systems in immune cells leads to selective immunosuppressant activity [73], decreasing autoimmune tendencies [74]. However, those with sustained hypovitaminosis D have less effective innate and adaptive immune systems. They have impaired second messenger signals and genomic stimulations through calcitriol–VDR interactions [62], resulting in hypo-responsivity of autoreactive T cells [75,76]. In contrast, when 25(OH)D concentrations are adequate, T-cell responsiveness is restored, and autoimmunity risks are reduced [72].

The active form of vitamin D calcitriol is essential for immunomodulating immune cells, such as monocytes, macrophages, dendritic cells, and T and B lymphocytes [76,77]. These cells express the enzyme CYP27B1 that hydroxylase calcifediol [25(OH)D] to calcitriol and vitamin D receptor (VDR)—the prime stimuli for activating the immune system [76,77]. These interactions produce anti-microbial peptides, such as cathelicidin and β-defensin 2 (see Section 3.6). Furthermore, in infected cells, calcitriol also increases the expression of nucleotide-binding oligomerization domain-containing protein 2, which damages the cell membranes of bacteria and viruses by activating signaling cascades [72]. 

In addition to anti-microbial peptides, calcitriol induces autophagy and gap protein [78] with tight gap junctions [79]—strengthening the integrity of epithelial and endothelial cells and preventing viral penetration and fluid leaks [80]. Vitamin D also enhances the expression of angiotensin-converting enzyme-2 (ACE-2) [81], suppressing the renin–angiotensin system and dampening inflammation [82]. Increased expression of soluble ACE-2 neutralizes circulatory viruses by binding to them, thus preventing SARS-CoV-2 from binding to cell membrane-bound ACE-2 receptors and cellular entry [83,84]. 

### 2.3. Vitamin D and Immune System

Evaluation of epidemiological studies illustrates that vitamin D deficiency increases susceptibility to infections and autoimmunity [62,64,66,85] and acquired autoimmunity [63]. 1α-hydroxylase (CYP27B1) and VDR are expressed in all immune cells, including by neutrophils, lymphocytes, dendritic cells, macrophages (antigen-presenting cells), and B lymphocytes, CD4+, and CD8+: these are stimulated when pathogens and foreign antigens are detected by Toll-like receptors-4 (TLR-4) [85,86].

With such intracellular signaling and sufficient quantities of calcitriol, they accelerate the 1α-hydroxylation of 25(OH)D to 1,25-(OH)_2_D and the synthesis of VDR. Calcitriol then suppresses the transcription of inflammatory cytokines and blocks IgE-mediated mast cell degranulation. The latter is one of the mechanisms to alleviate hives, allergic reactions, and disorders that exacerbate inflammation [72]. In contrast, vitamin D adequacy stabilizes mast cells, suppressing the release of histamine and TNF-α [62]. 

Vitamin D modulates several types of immune cells, including monocytes/macrophages, dendritic cells, and B and T cells [71]. Hypovitaminosis D increases vulnerability to inflammatory diseases and disorders with an autoimmune element, such as lupus, metabolic syndrome, and T1D [87,88,89,90,91] (see below). Following supplementing with vitamin D, clinically meaningful disease risk reductions have been reported in persons with MS, chronic fatigue syndrome, Behcet’s disease, inflammatory bowel diseases [77,92,93,94], and rheumatoid arthritis [95,96]. Still, not all study results agree [97,98]. 

In addition to the anti-inflammatory effects of vitamin D on T-helper cells, B cells, macrophages, and dendritic cells, vitamin D has broader immunomodulatory actions on innate and adaptive immune responses [99,100]. Regulation of immune responses by calcitriol partially inhibits B-cell expression of IgE and increased expression of IL-10 via dendritic cells and T cells [75,101,102,103]. Many of the above-mentioned biological functions occur following the genomic effects of calcitriol. The following section discusses some non-genomic effects of calcitriol on the immune cells.

### 2.4. Vitamin D Is Fundamental to the Defense against Microbes and Preventing Autoimmunity

Vitamin D deficiency leads to a dysfunctional immune system, creating increased susceptibility to bacterial infections, mainly intracellular bacterial infections [104,105], such as mycobacteria tuberculosis [106,107], and a variety of viral infections, including influenza A [40,105,108] (Table 1). In contrast, adequate circulating 25(OH)D concentrations are associated with decreased incidences of infections [109], enhanced immunity, and improved ability to overcome bacterial and viral infections [105,110]. Table 1 illustrates some examples of infections and autoimmune disorders improved with adequate serum 25(OH)D concentrations, demonstrating multiple mechanisms; calcitriol combats pathogens [24,111] and prevents autoimmunity [32,33] (Table 1).

## 3. Mechanisms of How Vitamin D Controls Infections and Autoimmunity

Calcitriol functions as a hormone following secretion into the bloodstream from the kidneys. This alters the behavior of the cells involved in calcium–phosphate–bone metabolism, intestinal mucosal cells, and bone and parathyroid cells. Generally, the circulatory concentrations of vitamin D and 25(OH)D (in ng/mL) are approximately 900-fold higher than calcitriol (in pg/mL) [114]. Consequently, the diffusion of hormonal calcitriol from the blood to peripheral target cells is too little to influence their biological activity [115]. Thus, unsurprisingly, circulatory calcitriol has no clinical and evidentiary impact outside the musculoskeletal, parathyroid, and fat cells [115]. Consequently, peripheral target cells’ physiological activities depend on the synthesis of calcitriol intracellularly. In response to membrane-based signaling from TLR-4, immune cells increase intracellular synthesis of calcitriol and VDR, generating genomic functions [116]. 

Calcitriol down-regulates inflammation and oxidative stresses by suppressing inflammatory cytokines and enhancing anti-inflammatory cytokines’ synthesis via the abovementioned mechanisms [117]. The immunomodulatory effects of vitamin D include activation of immune cells such as T and B cells, macrophage and dendritic cells, and enhanced production of several antimicrobial peptides and neutralizing antibodies [35,36,37].

### 3.1. The Importance of Intracellular Generation of Calcitriol for Immune Cell Signaling

Approximately 75% of the innate [32] and over 50% of the adaptive [118] immune systems are driven by intracellularly generated calcitriol [26]. The average circulatory concentration of calcitriol is approximately 0.045 ng/mL. However, its diffusible free form is less than half in the blood [119]. Even though unbound (free-form) calcitriol is fully diffusible into target cells, it occurs in a low pico-molar range (much less than the minimum concentration needed). These minute concentrations are far below the threshold required to initiate intracellular signaling or genomic activity [115]. Whether such has any biological function is not yet known.

As per present data, calcitriol’s hormonal form is unlikely to impact intracellular biological signal transduction or genomic functions in immune cells. This is yet another reason to avoid using pharmacological doses of synthetic calcitriol in infections or to overcome autoimmune conditions. The correct approach is to provide appropriate higher amounts of the precursor—vitamin D (including an upfront loading dose if indicated) [120], except for oral administration of calcifediol in emergencies [24,26].

Intracellular calcitriol is critical for modulating genomic [121] and non-genomic activities such as signal transduction [122,123]. The non-genomic functions include the tightening of the gap-junctions [124] and autocrine (intracrine) and paracrine signaling [24,36,37]. Intracrine signaling is initiated following the detection of foreign proteins, microbes, etc., by a series of cell surface receptors. The most important is the membrane-bound (sensing/detecting) TLR-4 [125], which is also involved in the production of antimicrobial peptides [86]. Intermittent signals derived from TLR-4 lead to over-drive peak production of calcitriol and VDR in mitochondria/microsomes [126] (see below for details). 

Immune cells do not have active (energy-dependent) cell-membrane transportation mechanisms, such as megalin–cubulin; only diffusible low concentrations of calcitriol get into immune cells from circulation. Consequently, in addition to a smaller quantity via endocytosis, only the diffusible calcitriol can enter immune cells from the circulation. The estimated intracellular calcitriol concentration in active status exceeds 1 ng/mL—an estimated minimum intracellular concentration needed for initiating immune cell functions. However, the average circulating concentration of hormonal calcitriol is approximately 0.045 ng/mL [118], which is less than the 20-fold required for intracellular signaling [127]. Therefore, at equilibrium conditions in immune cells, the free hormonal form of calcitriol diffusing into immune cells (~0.02 ng/mL) is too little to activate their functions, such as intracellular signaling or binding with VDR, leading to gene transcription [118].

This is another reason pharmacological doses, such as one or more micrograms of calcitriol, have little beneficial effects in infectious diseases, including SARS-CoV-2, as shown in failed RCTs [128,129]. The exceptions are calcitriol/VDR-resistant syndromes, hypoparathyroidism, and chronic renal failure, where exogenous calcitriol is lifesaving. [130].

### 3.2. Intracellular Calcitriol Signaling

When immune cells detect external threats, such as circulating antigens by key innate immunity pattern recognition receptors such as membrane-bound TLR-4 [131], they send signals to increase the expression of 1α-hydroxylase and VDR [118] and microsomal-apparatus to increase cytoplasmic concentrations of both [117,127]. As a result, immune cells synthesize higher (low nmol range) concentrations of non-hormonal calcitriol and VDR *in situ* [115,118]. This results in generating peaks of calcitriol in the cytosol, driving autocrine and paracrine signaling [36,37,114,127] and calcitriol/VDR complexes (not from the circulatory hormonal calcitriol) [24,132,133]. As described above, the latter does not enter meaningful amounts into peripheral target cells. (See Section 4.4 for more detail).

This provides a physiologically balanced intracellular autocrine/intracrine signaling, crucial for immune cell functions [118]. This critical early warning TLR-detection system evolved to identify and overcome threats from infection (or foreign antigens) and autoimmune responses. Since this is also a threshold mechanism, further increasing serum 25(OH)D concentrations (i.e., beyond 60 ng/mL) would not produce additional beneficial immune cell functions from an infection's point of view. 

When no external signaling exists, calcitriol and VDR synthesizing revert to a baseline steady state. This is an efficient evolutionary mechanism to stimulate immune cells as needed, intermittently, as needed—when an external threat—detecting unfamiliar (foreign) proteins or antigens in the circulation or local tissues. This sporadic phenomenon ensures the formation of sufficient calcitriol-VDR complexes to modulate gene transcriptions and calcitriol for intra-cellular autocrine regulations when needed and enough intracellular concentration of calcitriol for internal signaling, as described in the next section. 

### 3.3. The Importance of Autocrine and Paracrine Signaling for Immune Cell Functions

TLR-4-mediated calcitriol synthesized within the immune cells also enhances the expression of anti-microbial peptides and antibodies [134,135]. The exact mechanism of stimulation of these pathways is unclear, but it is known to involve transcription factors C/EBPβ and the inhibition of NR4A2, an orphan receptor [136]. The regulation of the CYP27B1 gene (1α-hydroxylase enzyme) by a transcription factor promoter, NR4A2, is inhibited by C/EBP-beta. Furthermore, over-expression of C/EBP-beta decreases NR4A2 and CYP27B1 mRNA levels [136]. 

In contrast, FGF-23 counteracts the activity of the 1α-hydroxylase enzyme through FGF receptors in the presence of the co-receptor (an aging-related factor), Klotho [31]. The ablation of Klotho leads to over-expression of FGF23, which is consistent with Klotho deficiency [31]. This signaling also activates the mitogen-activated protein kinase (MAPK), but its role in CYP27B1 expression remains unclear [137].

When the circulating D and 25(OH)D are low and at insufficient concentrations to enter immune cells, it hinders the generation of intracellular calcitriol. One example of calcitriol intracrine signaling is switching T helper cell 1 (Th1) to T helper cell 2 (Th2) and Th17 to Treg cells, which transforms pro-inflammatory status to anti-inflammatory status [36,37]. This maintains the inflammatory statutes of Th1 and Th17 cells; severe viral infections such as SARS-CoV-2 in vulnerable people could initiate cytokine storms and the development of ARDS [138,139]. 

### 3.4. Mechanisms of Decreasing Inflammation with Vitamin D Adequacy

Vitamin D has anti-inflammatory, anti-oxidant, and anti-mitotic actions. In addition, it stabilizes endothelium and improves smooth muscle cell functions. There is a statistically significant inverse relationship between serum 25(OH)D concentrations of less than 21 ng/mL and higher serum C-reactive protein (CRP) levels (an inflammatory marker) [140], suggesting an essential anti-inflammatory effect of vitamin D in humans. This generalized anti-inflammatory effect is one of the critical reasons for the observed cardiovascular-protective effects associated with calcitriol [141,142].

Vitamin D signaling decreases inflammatory responses, including reduction of the expression of pro-inflammatory cytokine and mediators (e.g., cyclooxygenases; 5-lipoxygenase), as demonstrated by genome- and transcriptome-wide studies. It also modulates transcription factors, such as the nuclear factor kappa light-chain (NF-κB) of activated B cells that regulate inflammatory gene expression and reduce mitogen-activated protein kinases’ activation [91,99]. Calcitriol also downregulates cytokine production and the biosynthesis of pro-inflammatory cytokines in the prostaglandin pathway and through NF-κB [99]. These actions explain a strong association between low serum 25(OH)D concentrations and the many inflammatory diseases mentioned. Despite these findings, no vitamin D or analog has been used in adequately powered RCTs to test efficacy in controlling inflammatory conditions [99,100,143].

Increased local generation of calcitriol has been reported in those with diabetic foot ulcers. This is considered a physiological response to chronic inflammation and an attempt to enhance immunity in local tissues to combat infections [40,144] (see Section 3.6 for the effects of cathelicidin). However, such chronic inflammations (in this case, vitamin D deficiency-induced) will increase the risks for other disorders like myocardial infarction and stroke. Moreover, an increased intake of micronutrients during periods of high stress reduced inflammatory processes and plasma lipids, particularly in males [145]. This may also have clinical relevance for those with diabetic foot ulcers and other chronic infections. These data support vitamin D’s important immunomodulatory and anti-inflammatory roles [144]. Examples of these are discussed below. 

### 3.5. Anti-Microbial Activities of Vitamin D 

Mycobacteria and/or activation of macrophages leads to enhanced intracellular 1α-hydroxylase activity within macrophages (e.g., in granulomatous tissues), leading to the generation of 1,25(OH)_2_D_3_. These increases in intracellular calcitriol accompany the increasing expression of the VDR in macrophages., as a defense mechanism in those with sufficient vitamin D status. Because this activity is not subjected to feedback control, if not intervened in a smaller number of patients, in some, it may increase serum concentrations of 1,25(OH)_2_D_3_, leading to (granuloma-related) hypercalcemia. 

The seasonal peaks of influenza have been attributed to a higher prevalence of vitamin D deficiency during the winter months [146]. Thus, persons with hypovitaminosis D are more susceptible to viral infections [62]. This hypothesis is supported by two recent RCTs: one was in black postmenopausal women with a baseline mean 25(OH)D concentration of 48 nmol/L [147], and the other was in a group of schoolchildren in Japan with low serum vitamin D [148]. In the latter, daily supplementation with 1000 IU of vitamin D_3_ significantly reduced the risks of type-A influenza by two-thirds but did not affect type-B influenza. 

Moreover, a meta-analysis of 11 RCTs on vitamin D supplementation concluded that once-daily dosing with vitamin D supplements had a significantly better response rate than did intermittent dosing regimens, such as monthly dosing (odds ratio = 0.51 vs. 0.86; *p* = 0.01) [149]. Those who experience pneumonia also had an increased prevalence of vitamin D deficiency [150]. Low serum 25(OH)D concentration is associated with low cellular immune functions and an increased risk for hyponatremia, as reported with H7N9 pneumonia [151]. However, the relationship between these two entities is unknown, and not all RCTs support this concept [152,153].

### 3.6. Vitamin D Enhances the Expression of Bactericidal Proteins

T cells and macrophages both have a high concentration of VDRs [71,154]. Vitamin D—receptor interactions increase the expression of potent bactericidal and viricidal protein cathelicidin, which combats mycobacterium organisms, such as tuberculosis and lepra, and other intracellular bacteria [107]. In addition to cathelicidin, VDR activation increases the synthesis and secretion of multiple other bactericidal peptides, including defensins [155,156]. Thus, calcitriol adequacy, while reducing the expression of inflammatory cytokines, also increases the secretion of bactericidal peptides in vivo [141,157], complementing the immune system to combat invading microbes. 

Although individual studies indicate positive results, when data from multiple microbiological studies are pooled, the results may not necessarily lean in a positive direction because of the heterogeneity of studies used in meta-analyses. Inclusion of studies that differ concerning population, ethnicity, age range, types of infections, severity, study design, and duration, mode of observation or randomizations, and baseline serum 25(OH)D concentrations and/or the serum concentrations achieved have muddled the situation and led to inaccurate conclusions.

Vitamin D reduces risks for and the spread of chronic infections [10,11,13], particularly mycobacterium tuberculosis, by regulating innate and adaptive immunity. Sufficient amounts of intracellular calcitriol in immune cells augment innate responses (monocytes/macrophages with anti-microbial activity) and suppress adaptive immunity (T- and B-lymphocyte activities) [61]. In addition, calcitriol modulates B lymphocytes, immunoglobulin production, and B-cell homeostasis [61]. 

Because of the variability of studies and poor study designs, vitamin D dosing, and recruitment, the pooled RCT data from a vitamin D, many meta-analyses cannot be relied upon [158]. Thus, to generate meaningful conclusions, future RCTs should be focused on subjects with documented vitamin D deficiency measured at recruitment to confirm low serum 25(OH)D concentration and subjects in the treatment arm provided with adequate vitamin D supplements to achieve a predefined target serum 25(OH)D concentration (but prohibited from taking over the counter nutrients in both arms), and standardized measurable hard outcomes.

### 3.7. Multiple Sclerosis and Autoimmune Encephalomyelitis

Without supplements, serum 25(OH)D concentrations can be a reliable surrogate marker of UVB exposure. However, there are additional, non-vitamin D-related beneficial effects of UVB exposure, such as reductions in the severity of depression and the risk of MS [159,160,161,162], of which mechanisms are ill-understood. In addition, exposure to UVB potentiates the suppression of experimentally induced autoimmune encephalomyelitis in animal models [163,164]. In people with MS, low 25(OH)D concentrations are an independent, positive predictor of disease progression [165]. Furthermore, a better response has been reported with interferon beta (IFN beta) in those with higher serum 25(OH)D concentrations [166,167].

In persons with MS, serum adipocytokine concentrations are positively correlated with inflammatory mediators and negatively correlated with Foxp3 expression [168]. In that study, positive correlations were also reported between leptin and resistin concentrations with TNF-alpha and interleukin 1β (IL-1β), with the highest levels of TNF-alpha, IL-1β, CRP, resistin, and leptin reported in persons with progressive MS [168]; some of these are positively modulated by calcitriol.

Overall data suggest a clinically meaningful suppression of autoimmune disorders when serum 25(OH)D concentrations are maintained at greater than 40 ng/mL [5,31,112], preferably over 50 ng/mL (range 50 to 80 ng/mL). Supporting this, a longitudinal, prospective observational study by the author demonstrated that in those with chronic MS (n = 64), keeping serum 25(OH)D concentrations above 40 ng/mL over an average 2-year period resulted in an 80% reduction in recurrences (i.e., reactivation rate) [31].

### 3.8. Autoimmunity—Rheumatoid Arthritis and Lupus

As with calcitriol therapy, supplementation with high doses of vitamin D improves cell-mediated immunity [169]. One study reported that monthly administration of 140,000 IU of vitamin D_3_ over three months significantly increased the regulatory T-cell population in healthy people compared with the administration of a placebo. Similarly, doses of vitamin D_3_ (4000 IU daily) significantly decreased CD4 cytotoxic T-cell activation compared with low-dose vitamin D_3_ (400 IU/day) therapy [170].

Another study using vitamin D, 10,000 IU/day over six months, reported favorable immunomodulatory effects, including suppression of IL-17 production and improvement in the effector CD4+ memory cells, with a concomitant increase in central memory CD4+ cells [171]. These data confirmed that vitamin D supplementation effectively elevates circulatory 25(OH)D concentrations in persons with inflammatory diseases and vitamin D deficiency, benefitting them [172].

In vitro, animal studies have suggested that 25(OH)D and 1,25(OH)_2_D have independent immunomodulatory effects. However, cell culture and animal studies use micromolar concentrations of calcitriol (i.e., about 1000-fold higher concentrations than present in humans), thus should not extrapolate to humans. In a study of patients with arthritis, a decrease in the Disease Activity Score-28 and a 25% reduction in serum CRP levels occurred with each 10-ng/mL increase in serum 25(OH)D concentration [173], demonstrating a powerful and protective anti-inflammatory effect of vitamin D, as in the case of rheumatoid arthritis [174]. 

Those with genetic resistance to calcitriol, a rare genetic disorder, have a higher incidence of autoimmune diseases such as rheumatoid arthritis [175]. Therefore, patients with rheumatoid disorders also benefit from vitamin D repletion. A recent study suggested that an acquired tissue resistance to calcitriol in those with rheumatoid arthritis may require vitamin D analogs [176]. In addition, those exposed to adequate doses of UVB reduced their complications and progression of rheumatoid arthritis [61,177,178]. Nevertheless, post hoc analysis of the Women’s Health Initiative study failed to show an association between rheumatoid arthritis and solar irradiation [179]. However, such piggyback studies and secondary analyses are neither designed nor statistically powered to address such issues.

## 4. Improving Clinical Outcomes via Vitamin D Sufficiency

When circulating D_3_ and/or 25(OH)D is adequate (e.g., over 50 ng/mL), these precursors diffuse into peripheral target cells, such as immune cells, in adequate quantities from the circulation. This process allows the intracellular generation of sufficient calcitriol for signaling and genomic activity. Calcitriol suppresses the pathological process and hyper-immune reactions with its genomic actions and autocrine signaling mechanisms [36,37]. These actions reduce the risks of cytokine storms and ARDS and are associated with severe pulmonary and cardiovascular complications in persons with severe infections such as COVID-19 [180,181].

### 4.1. Importance of cofactors and micronutrients for the full functions of vitamin D 

The full activity of vitamin D, VDR, and associated enzymatic reactions require either the endogenous presence, or the administration of several cofactors [182,183]. These include magnesium, vitamins A, B_2_, C, and K, anti-oxidant trace minerals (zinc and selenium), resveratrol, essential fatty acids such as omega-3, and boron [3,184]. Besides, the functioning of the immune system and other target cells continues to consume vitamin D and its metabolites and cofactors [41]. This requirement is enhanced due to the multiple immune and metabolic pathways in which vitamin D is intimately involved in vivo. 

Consequently, a continuous supply (preferably daily intake during an illness) of the mentioned micronutrients is necessary to attain optimal potentials of vitamin D and better clinical outcomes [41,183]. The lack of this is another reason for the little benefits reported in some clinical studies, including RCTs. This is important in both acute and longer-term clinical trials. In clinical studies, including RCTs, and clinical practice, scientists/physicians have ignored this critical factor (considered as another study design error). At the minimum, study subjects (active and placebo participants) and clinical patients should be provided a multivitamin and essential mineral supplements (e.g., magnesium, zinc, selenium, boron, etc.) during an illness [182,183,184], enabling them to recover faster.

### 4.2. Consequences of Hypovitaminosis D

Severe vitamin D deficiency is associated with immune dysregulation. Consequently, when subjected to a severe infection, they could develop hyperinflammation, oxidative stress, and autoimmunity [85,185,186]—an over-reactive pathological immune response [187]. The failure to correct vitamin D deficiency rapidly could lead to cytokine storms with an increased risk of death [188,189]. This could precipitate acute respiratory distress syndrome (ARDS) in severe respiratory tract infections [62] and asthma [66]. This is preventable with an appropriate dose and type of vitamin D (e.g., calcifediol instead of vitamin D_3_). 

Due to the impairment of the formation of intracellular calcitriol in the immune cells, hypovitaminosis D also impairs intracrine and paracrine signaling, further weakening the immune system, and increasing vulnerability [37,190]. Cytokine storms are associated with pro-inflammatory and hyper-oxidative stress responses in severe viral infections. This increases intensive care unit (ICU) admissions and the risk of death, as observed during the COVID-19 pandemic [48,49,50,51]. Children infected with SARS-CoV-2, having less than 12 ng/mL of serum 25(OH)D concentrations (i.e., severe vitamin D deficiency), are at very high risk for developing life-threatening hyper-inflammatory conditions, such as Kawasaki-like disease or multi-system inflammatory syndrome [191,192,193].

Moreover, hypovitaminosis D leads to weakened adaptive immunity, which reduces the capacity to generate neutralizing antibodies (including after vaccination) and impairs the cytotoxic action of immune/killer cells. It also reduces the effectiveness of memory cells and macrophages and causes weaker responses following (any) vaccine. Overall, it causes immune paresis with inadequate antibody responses. 

In those with a fragile immune system, as in severe hypovitaminosis D, not only SARS-CoV-2 infection but also immunization against it could lead to significant adverse effects [194,195,196,197]. The latter include hyper-immune and autoimmune reactions, generalized hyper-inflammation, and pathological oxidative stress, which increase the risks for systemic complications (blood clots, strokes, etc.) and death. Consequently, in 2020/21, due to the prevailing high incidence of hypovitaminosis among older people and those with comorbidities, COVID-19 primarily affected them, sparing children, and the youth [2,3]. However, the vaccine-related adverse effects continue among those with hypovitaminosis D.

### 4.3. One Serum 25(OH)D Concentration Would Not Control All Diseases

Different diseases require varying serum 25(OH)D concentrations to obtain the best clinical outcomes and prevent complications [5,31,112]. Many conditions require maintaining serum 25(OH)D concentrations greater than 30 ng/mL for anticipated clinical outcomes. There is no one optimal serum 25(OH)D concentration that provides maximum beneficial outcomes for all body systems [198,199]. While the musculoskeletal system may benefit from lower levels of approximately 20 ng/mL, other body systems require more than 40 ng/mL. Examples include T2D and metabolic syndrome [200,201]. However, alleviating others, such as cancer [202], asthma [66], autoimmunity, infections, and cancer [31,203], etc., requires the maintenance of serum 25(OH)D concentrations greater than 50 ng/mL (See Section 4.4 for more details). 

Dark-skinned people in central Africa living traditional lifestyles have a mean serum 25(OH)D concentration of 47 ng/mL (119 nmol/L) (range, 30 to 70 ng/mL) [204,205]. However, with imbalanced macro-nutrient diets, micro-nutrient deficiencies, unwholesome modern dietary constituents and practices (e.g., unhealthy processed food, fast-food, trans fat, and preservatives, some of which also increase the catabolism of micronutrients), environmental pollution, and passive indoor lifestyles, many people likely require much higher vitamin D intakes than those recommended by governments and health-related societies and need to maintain a higher range of serum 25(OH)D concentration, such as 50 to 80 ng/mL to obtain vitamin D-related benefits. 

### 4.4. Minimum and the Range of Serum 25(OH)D (ng/mL) Necessary to Minimize Diseases and Obtain Maximum Benefits

Considering broader biological and physiological fundamentals, changing disease patterns (metabolic diseases, obesity, diabetes, and increasing prevalence of viral infections), the behavior of people (sun avoidance), and broader risk factors (pollution, harmful diets, medications, etc.) [206], and passive lifestyles acquired in this Millenium [207], the published evidence justifies the above-mentioned higher range of serum 25(OH)D (50 to 80 ng/mL). Based on the data, it is also reasonable to contemplate that the minimum serum 25(OH)D concentration needed for a healthy life for all ages of humans is 50 ng/mL.

In addition, evidence strongly suggests that there are tissue-specific differences in serum 25(OH)D concentration thresholds to elicit full biological effects. The previously suggested minimum serum level of 25(OH)D—30 ng/mL—would only protect less than a third of common disorders (primarily calcium homeostasis and musculoskeletal). In contrast, the one suggested above—50 ng/mL—is the minimum adequate level (with a range of 50 to 80 ng/mL) and would cover 99% of health conditions with no adverse effects. 

In contrast, if one considers 80 ng/mL as the minimum level (as some indicated), it will cover 99.8% of health conditions but is likely to increase adverse effects; thus, it is not justified or recommended. Data supports the idea that less than 0.01% of the population requires very high doses of vitamin D with a high response rate (see Section 4.4). Examples include prevention of intractable migraine headaches, asthma, psoriasis, specific autoimmune reactions and diseases, tissue/organ graft rejection, and vitamin D-resistant syndromes. These persons must be treated by specialists in this field (not general specialists, including immunologists and endocrinologists) under their close medical supervision to maximize benefits and minimize adverse effects. As per common sense and medical ethics, healthcare workers must strike a safe and cost-effective approach, the correct dose for a given person (individualized therapy), and a condition to obtain maximum benefits while avoiding adverse effects. Precautionary steps are taken with higher daily vitamin D doses (e.g., above 7000 IU/day or 50,000 IU/week) to prevent potential soft tissue calcification. These include avoiding calcium supplements and high calcium-containing food and taking vitamin K_2_ (MK-7: Menaquinone-7, present in fermented food), 100 micrograms/day or 800 micrograms, once a week.

Since having physiological serum 25(OH)D concentrations can control several acute and chronic conditions [208], it is logical to aim to maintain a population serum 25(OH)D above 40 ng/mL [115], and the concentrations of individuals to above 50 ng/mL [24,26]. To benefit the population and to reduce all-cause mortality, doubling the current prevailing population serum 25(OH)D concentration of approximately 20 ng/mL is needed [209,210]. This would mitigate the ongoing low-grade inflammation and chronic diseases in the population and open doors to obtaining broader benefits from vitamin D, such as controlling inflammation and oxidative stress [85], including reducing myocardial infarctions and strokes. It can enhance cellular effects such as membrane stabilization, protection from DNA damage (and repair), and minimizing infectious outbreaks and sepsis. 

### 4.5. Vitamin D Intakes and Optimum Circulating 25(OH)D Concentrations Needed to Overcome Diseases

Vitamin D and 25(OH)D have a high affinity to VDBP. This provides the means for transporting and increasing the half-life of 25(OH)D in circulation [115]. In addition, some cells, such as renal tubular cells, fat, and muscle cells, have an evolutionary mechanism of the active transportation of compounds such as vitamin D bound to VDBP. This megalin–cubulin mediated membrane internalization of vitamin D and 25(OH)D molecular complexes bound to VDBP provides an energy-dependent entry mechanism for these molecules into renal, parathyroid, fat, and muscle cells. This active transportation offers the generation of calcitriol in proximal renal tubular cells and storage of the nutrient in others [211,212].

This entry facilitates the synthesis of the hormonal form of calcitriol in proximal renal tubular cells, work via calcium-sensing receptors in parathyroid cells in conjunction with parathyroid hormone (and negative control by FGF-23) [115], maintaining the calcium homeostasis via modulating bone resorption and intestinal calcium absorption, and the renal tubular reabsorption of calcium [115]. In contrast, muscle and fat cells have the mentioned active cell membrane-based transportation system for storage for D_3_ and 25(OH)D transfer. Unlike pharmaceuticals, these active mechanisms prolong the half-life of 25(OH)D [1,38]. For administered vitamin D and serum 25(OH)D concentration, dose-response is not linear [213,214,215,216]. 

**Figure 4:**
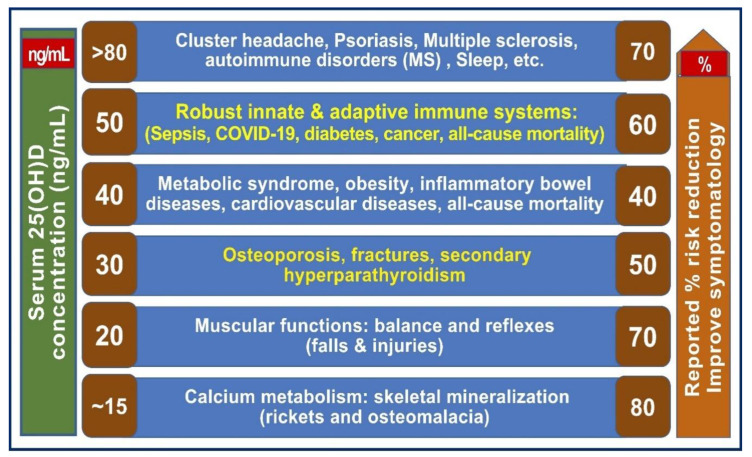
Different diseases (and tissues) require different steady-state serum 25(OH)D concentrations to achieve improvement: the need for varied serum 25(OH)D concentrations to subdue various disease statuses is illustrated (modified from Wimalawansa, S.J. Steroid Biochemistry [31].

Minimum serum 25(OH)D concentrations are needed to prevent or lessen the effects of common diseases. Figure 4 indicates the relationships between various disease states and the approximate minimal serum 25(OH)D concentrations needed to improve different conditions [31]. It summarizes the varying steady-state serum 25(OH)D concentrations required to prevent or lessen the effects of common diseases based on many published data.

### 4.6. Vitamin D Dose-Responses

**Figure 5:**
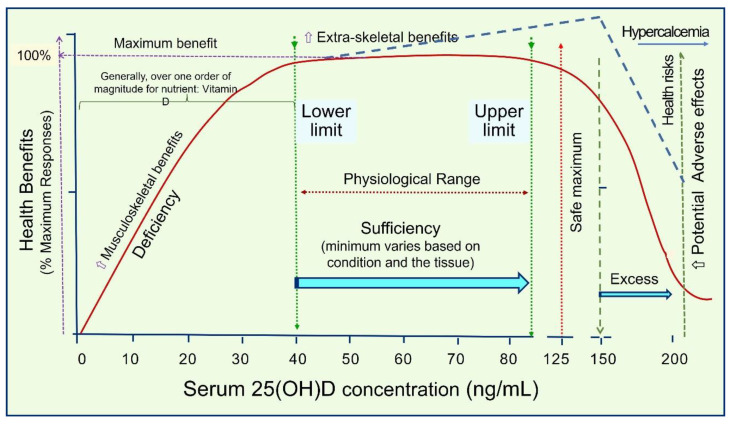
Illustration of the dose/25(OH)D concentrations achieved in the circulation vs. responses (clinical health benefits and potential risks). It also provides the basic pharmacodynamics of a typical nutrient, taking vitamin D as an example. When tissue sufficiency occurred, generally, there would not be additional benefits by raising the circulatory concentration by increasing the intake. However, there are exceptions in a small percentage; pharmacological doses are needed under medical guidance in less than 0.01% of the population to overcome resistance to achieve the desired clinical goals (indicated in the dashed blue line) [115].

Administration of high oral doses of nutrient vitamin D in D-deficient persons leads to a meaningful, measurable change in the serum 25(OH)D concentrations within three to four days [115,215,216]. The lower the serum 25(OH)D concentration, the higher the percentage increase (∆) in the circulation and the higher the likelihood of demonstrating a significantly better clinical outcome. However, such a dose-clinical response relationship does not exist in those who are vitamin D sufficient. Figure 5 illustrates a typical dose-clinical response curve for nutrients such as vitamin D. 

The current study strongly suggests that most health benefits are seen when serum 25(OH)D concentrations are maintained at more than 40 ng/mL (100 nmol/L) [217], with further improvements seen when levels kept over 50 ng/mL [24,115]. This can be cost-effectively achieved by providing safe sun exposure guidance and appropriate intakes of vitamin D supplements. The emphasis is on sensible, safe, balanced vitamin D (and other micronutrient intake) that provides cost–benefits to the public [218,219,220]. 

## 5. Other Considerations with Vitamin D

### 5.1. Adverse Effects of Vitamin D Are Rare

Vitamin D toxicity rarely manifests after consuming very high amounts (e.g., intake of above 20,000 IU/day by a non-obese 70 kg person) for prolonged periods. It has been demonstrated that daily oral vitamin D doses of up to 10,000 IU are safe and devoid of adverse effects. Reported data suggest that daily doses greater than 40,000 IU can harm individuals with normal calcium absorption profiles. However, a few patients with morbid obesity, gastrointestinal absorption issues, or those who are vitamin D resistant might need higher daily doses. The numbers requiring such remain very small. 

While adverse effects are rare, the few reported cases of vitamin D toxicity were due to mistaken doses or accidental use. Because of the built-in feedback control mechanisms within the skin, excessive exposure to UVB from sunlight does not cause vitamin D overproduction. These rescue catabolic pathways will divert the vitamin D metabolism to inert compounds such as 24(OH)D, 24,25(OH)_2_D, and other inactive metabolites [221]. Nevertheless, excessive sun exposure without protection is not recommended, as it could increase the risk of skin damage and potential malignancies but would not result in vitamin D toxicity [6,30].

Hypervitaminosis D-induced toxicity should not be diagnosed solely based on elevated 25(OH)D levels. Instead, it should be recognized as a clinical syndrome in the presence of hypercalcemia, suppressed PTH, and hypercalciuria in conjunction with markedly elevated serum 25(OH)D levels (>150 ng/mL). The rare occurrence of vitamin D-related symptomatic adverse effects, such as hypercalcemia and hypercalciuria, could result from individuals taking extremely high doses of vitamin D (especially activated vitamin D analogs) for a prolonged time or taking large amounts inadvertently. The clinical signs and symptoms of vitamin D toxicity include hypercalcemia (e.g., nausea, dehydration, irritation (dryness) of the eyes, confusion, constipation, and electrocardiographic abnormalities), irritability, and hypercalciuria (e.g., polyuria and kidney stones).

Asymptomatic elevation of 25(OH)D without hypercalcemia needs to be investigated for the etiology of increased vitamin D levels. Unlike hypercalcemia (i.e., higher ionized calcium in the blood), increased vitamin D [25(OH)D] levels are not a medical emergency. If the issue is too much intake, it is essential to stop taking vitamin D supplements, including multivitamins. Lower doses of vitamin D supplements can be restarted once the 25(OH)D level reaches a low normal range with modification of the amount and diet. Most patients with vitamin D toxicity have serum concentrations greater than 150 ng/mL.

Data indicate that regimens of vitamin D supplementation with 10,000 IU/day or 50,000 IU bimonthly (even weekly) are not associated with laboratory or clinical variables of toxicity (manifested as serum calcium, bone alkaline phosphatase, and 24-h urine calcium), confirming the safety of such regimens [222]. Eleven patients with symptomatic hypercalcemia caused by hypervitaminosis D had taken supplement doses greater than 50,000 IU/day or 600,000 IU (injectable form) too frequently for various ailments, including back pains, osteoarthritis, or osteoporosis, for several months. Such toxicity is easily avoidable [223,224]. In rare occasions, macrophage-driven, autonomous production of 1,25(OH)_2_D may occur in granulomatous tissues. This is caused by a lack of feedback control of 1α-hydroxylase enzyme in granulomas, such as sarcoidosis and tuberculosis. It can cause hypercalcemic syndrome [225,226]. 

### 5.2. Personal Vitamin D Response and Targeted Serum 25(OH)D Concentrations

Standardized technology assesses vitamin D status by measuring serum 25(OH)D—the predominant circulatory and storage form [227]. Normal serum concentrations of 25(OH)D and 1,25(OH)_2_D are essential for optimal musculoskeletal and soft tissue health. However, the circulating physiologic calcitriol concentrations are unrelated to extra-skeletal health and, thus, do not affect the functions of peripheral target cells (such as immune cells). Consequently, what matters for extra-skeletal body systems is the ability to diffuse enough vitamin D and/or 25(OH)D into peripheral target cells, enabling them to function optimally. 

While a personal vitamin D response index may provide better guidance for optimizing vitamin D supplementation for individuals than broader population-based recommendations [228,229], its associated unnecessary cost and associated impracticality prevent its use. Such an index could be helpful only if performed inexpensively, like finger-stick blood sugar measurement [230]. Even if an index and testing provide theoretical benefits of a targeted increase of serum 25(OH)D concentration, such results may be difficult to sustain. 

Circulating 25(OH)D sufficient for target tissue cell activation of calcitriol (and VDR) allows beneficial modulatory effects on cellular functions, especially mitochondrial activity, enzymatic reactions, and hormone synthesis and secretion. Examples of the latter include insulin PTH, renin–angiotensin–aldosterone, and FGF23–Klotho system. In conjunction with adequately supplemented clinical studies, data from metabolomics and transcriptomics would provide better information on longer-term extra-skeletal benefits. In addition, adequate vitamin D supplementation allows personalized and targeted measures to reduce skeletal and soft tissue health risks cost-effectively [9,228]. 

### 5.3. Vitamin D Is a Threshold Nutrient

As with some others, vitamin D is a threshold nutrient; its beneficial effects can be demonstrated only in those deficient in vitamin D [215]. Unlike pharmaceutical agents, in those who are vitamin D sufficient, no matter how high the doses provided, there will not be additional benefits [115] (with very few exceptions discussed above). This view is supported by adequately powered and well-conducted RCTs in vitamin D-deficient subjects for the required duration; using proper amounts of vitamin D (daily or once a week) almost always provided positive results. 

Because vitamin D is a threshold nutrient (with different tissue sensitivities), the only way to demonstrate the intended favorable clinical outcomes in an RCT or a prospective clinical study is by conducting properly designed investigations in subjects with vitamin D deficiency [231,232,233,234,235]. Those tested for vitamin D deficiency-related primary clinical outcome(s) (i.e., testing a hypothesis—pre-determined health benefits) reported substantial benefits following the correction of vitamin D deficiency [236,237,238]. 

Empirical evidence establishes the connection between exposure and clinical outcomes. Clinical studies show that infections can be prevented by proactively correcting vitamin D deficiency in individuals who are vitamin D deficient and, in the community, [239,240,241]. In RCTs, with proper daily or once-a-week vitamin D supplementation in the intervention group, the serum 25(OH)D concentration must be meaningfully increased to a pre-planned level to ensure the validity of the clinical study. Instead of the administered dose, the serum 25(OH)D concentration achieved and maintained in the circulation (a pre-determined level) should be used for correlations with clinical outcomes (authentic dose responses), especially in longer-term studies. 

Well-designed and conducted clinical studies, as mentioned above, have reported a significant reduction in the risks of SARS-CoV-2 infection and complications [239,240,241]. Such will boost and maintain a robust immune system that lessens the risks from SARS-CoV-2 infection and its complications—altering the cause-and-effect and leading to better clinical outcomes (Koch’s postulates). This data provides strong evidence for a causal relationship between vitamin D and its physiological effects, as demonstrated in UK BioBank data [49,50,51]. 

### 5.4. Vitamin D Deficiency Increases Vulnerability to SARS-CoV-2 Infections

Evidence strongly supports that low vitamin D status increases the rates of infections, complications, and mortality rates for intracellular bacterial diseases such as tuberculosis and viral respiratory illnesses in both children [242] and adults [40,243], including from SARS-CoV-2 infection [231,232,233,234,235]. In addition, pre-existing vitamin D deficiency increases the risks of SARS-CoV-2 infection [239,240,241], its complications [240,244,245], hospitalizations [49,50,51,57,246], and deaths [48,59,245,247,248]. In contrast, proper doses, and frequency of vitamin D supplements in deficient persons significantly reduce risks for infections, complications, and deaths from SARS-CoV-2 [48,49,50,51,57,239,240,241,244,245,246,247,248]. 

Reported data validate Bradford Hill’s criteria for causation of diseases [60]: vitamin D deficiency causing cancer [249,250,251], multiple scleroses [252,253], the risk of contracting SARS-CoV-2 infection [46,47,51,239,240,254], and the severity [255], and the vulnerability and complications for SARS-CoV2 [231,232,233,234,235,239,240,241]. Further, the crucial mechanism of action of intracellular calcitriol in immune cells supports the biological plausibility that low vitamin D increases the risks for infections, including SARS-CoV-2 [53,56,247,251,255,256,257]. In addition, data demonstrated that vitamin D significantly reduces complications and deaths from SARS-CoV-2 [32,48,49,50,51,56,57,239,240,241,244,245,246,247,248,257].

### 5.5. Issues with Published RCTs and Limitations of Data and Interpretation

Adequately powered, well-designed epidemiological studies and RCTs that used adequate doses of vitamin D supplementation to achieve a predefined target serum 25(OH)D concentration in subjects with hypovitaminosis D reported favorable outcomes. Such studies have demonstrated the importance of maintaining an optimum serum 25(OH)D concentration for normal physiologic functions and improved quality of life. While in some areas, definitive evidence is lacking, it is mainly due to published RCTs with major study design errors. The overall data support the protective effects of vitamin D in humans when 25(OH)D serum concentration is maintained above 50 ng/mL [115]. From the practical and community’s point of view, the goal for sufficiency should be above 40 ng/mL to achieve a balance.

Despite the above, there is little evidence from RCTs regarding the optimum serum 25(OH)D levels for preventing various disease-related complications. This confusion derives from the non-standardized, poorly designed clinical studies using different serum 25(OH)D concentration targets or no targeted serum 25(OH)D concentrations and attempted to correlate clinical outcomes with administered dose than with what achieved (or effective) circulatory concentrations [258]. These confusions partly derived from failing to understand that vitamin D is a threshold nutrient [214,215,259], not a synthetic pharmaceutical agent. This major misunderstanding exists even in extensive and expensive, public-funded vitamin D RCTs and almost all pharma-designed vitamin D-related RCTs, as they have done for pharmaceutical agents [115,215].

Irrespective of the number of participants enrolled in recent RCTs, as in the case of the VITAL study [260,261], it led to disarray because of poor study design [26]. Before studies commenced, these errors were pointed out to the NIH—the funding agency, but they failed to rectify them. Adequately powered studies with an appropriate format and suitable study duration recruited 25(OH)D deficient participants. The target serum 25(OH)D concentrations achieved and maintained during the RCT allow proper testing of specific vitamin D-related hypotheses [115]. While such studies are not so frequent, they have ubiquitously demonstrated the protective effects of vitamin D [262]. 

Future clinical studies must target predefined serum 25(OH)D concentrations for statistical correlations and use vitamin D supplementation as the only (or at least as the key) intervention to address vitamin D-related risk reductions as the primary hard endpoint specifically. Despite the accumulating data, awareness lags behind the beneficial effects and the optimal serum 25(OH)D concentrations concerning humans in non-musculoskeletal diseases [5,30]. Disagreements abound regarding optimal serum 25(OH)D concentrations, recommended oral supplementation doses, properly designed and adequately powered randomized clinical studies (RCTs), and outcome data. Nevertheless, it is fruitless to repeat the jargon that ‘more RCTs are necessary to conclude’ should not be included in metanalyses. If the studies are insufficient, authors should not have done such repeats of meta-analyses. 

### 5.6. New Vitamin D Recommendations 

Individual countries and scientific societies need to re-assess vitamin D guidelines to raise the recommended dietary allowance (RDA) of vitamin D, including higher amounts for food fortification guidelines and new targets to achieve better health for the public, as described above. Studies reported from Western Europe suggest that the use of such approaches may reduce the economic burden of common medical disorders, such as type 2 diabetes (T2D), cardiovascular diseases (CVDs), and cancer [263].

Steady-state serum 25(OH)D concentrations primarily depend on the body weight (BW) and the total fat mass. While body mass index (BMI) (validated only for White Caucasians) is not a good indicator of body fat estimation in ethnic groups like Asians. However, it is a helpful indicator encompassing fat and muscle mass, is readily available. Therefore, for calculating vitamin D dose for individuals, one can use either the BMI or the body weight, as illustrated below [24]. These simplified calculations are based on the detailed tables published in Nutrients in 2022 [Wimalawansa, SJ, Nutrients, 14(14), 2997, 2022; https://doi.org/10.3390/nu14142997] [24]. The following summarizes vitamin D dose calculation for an individual, applicable across all body weight groups.

I. Not obese (average wt.: BMI, <29): 70–90 IU/kg BW
II. Moderately obese (BMI, 30–39): 100–130 IU/kg BW
III. Morbid obesity (BMI, over 40): 140–180 IU/kg BW

All current vitamin D guidelines are based on decades-old concepts and research; they are outdated. Based on recent data, raising the minimum and maximum serum 25(OH)D concentrations to 50 and 80 ng/mL, the safe upper limit of intake to 15,000 IU/day, and the average daily intake of vitamin D, recommendation based on 5000 IU for a non-obese 70 kg adult (70–90 IU/kg body weight) is logical. Such will significantly reduce disease and hospital burdens, healthcare costs, loss of productivity and absenteeism. 

## 6. Discussion 

This systematic review examined the effects of vitamin D beyond calcium homeostasis and the musculoskeletal system. The current paradigms related to vitamin D are primarily based on retrospective analyses, case reports, and epidemiological studies (cohort, cross-sectional, observational, prospective, and ecological studies) [5,31,112]. However, an overwhelming number of reports support the positive effects of vitamin D outside the musculoskeletal body systems [218,220,264,265,266]. 

The knowledge of the physiology of D_3_ and vitamin D–VDR has advanced the understanding of the biology, metabolism, and effects of gene polymorphisms on the vitamin D axis. During the past decade, many advances have been made in understanding the physiology and biology of vitamin D, and its receptor ecology has emerged. Notably, a minimum serum 25(OH)D concentration of 50 ng/mL is crucial for immune cells and other extra-musculoskeletal target cell physiological activity. The lack of inclusion in current vitamin-related recommendations makes them outdated—another reason why guidelines must be updated. 

Evidence supports strong physiological associations of vitamin D with disease risk reduction and improved physical and mental functions. Together, these data have facilitated our understanding of new pathways for intervention to prevent and treat human diseases cost-efficiently. Overall evidence suggests that vitamin D deficiency, as determined by maintaining serum 25(OH)D concentrations of more than 40 ng/mL, is associated with increased risks of illnesses and disorders and higher all-cause mortality, even among otherwise healthy individuals [259]. The proper functioning of the vitamin D endocrine, paracrine, and autocrine systems is essential for many physiological activities and for maintaining good health. This systematic review addressed key functions of vitamin D that extend beyond its calcium and phosphate homeostasis and prevention and treatment of rickets, osteomalacia, and bone loss.

Recent data from epidemiological, cross-sectional, and longitudinal studies support that having physiological serum concentrations of 25(OH)D, levels greater than 40 ng/mL, significantly reduces the incidence of extra-musculoskeletal disorders. The latter includes diabetes [267,268,269], MS [270], rheumatoid arthritis [271], osteoporosis [272,273], autoimmune diseases [274], and certain types of cancer [275,276,277,278], as well as reducing all-cause mortality [259].

The dosages of vitamin D prescribed for non-obese deficient persons of average weight of 70 kg should be between 4000 and 7000 IU/day, 20,000 IU twice a week, or 50,000 IU once a week or once in 10 days [115]. Such doses would allow approximately 97.5% of people to maintain their serum 25(OH)D concentrations above 40 ng/mL [5,30,204]. However, intermittent doses at intervals longer than once a month are unphysiological and thus ineffective [279,280]. Studies have shown that daily vitamin D supplements are more beneficial than supplementation administered less frequently [281,282,283,284,285].

Furthermore, many medications, environmental pollutants, and physical activities/lifestyles influence vitamin D metabolism and actions, modulating the balance between energy intake and expenditure [286]. The mentioned factors should be incorporated into guidelines, future RCT study designs, and clinical practice. 

In contrast, using vitamin D analogs is inappropriate for alleviating hypovitaminosis D or treating osteoporosis [115]. In the absence of adequate exposure to sunlight, average-weight non-obese individuals require daily vitamin D intake (food plus supplements) of between 5000 and 7000 IU to maintain serum 25(OH)D concentrations above 50 ng/mL (125 nmol/L). Longer-term maintenance of a steady state of the serum 25(OH)D concentration is necessary to have a meaningful impact on reducing disease incidences and all-cause mortality [287]. 

This study confirms the need to combine approaches to alleviate vitamin D deficiency. Such strategies include enhancing awareness, fortifying food, advocating safe sun exposure, and vitamin D supplementation. Clinical practice recommendations should be geared toward healthcare professionals and the public, guiding patient education, and informing the public regarding appropriate actions for avoiding micronutrient deficiency. However, most countries neither have policies or guidance on sun exposure and vitamin D intake nor cost-effective public health interventions, especially for micronutrients. They should consider embracing cost-effective measures to prevent diseases, significantly reducing healthcare costs. 

Vitamin D deficiency increases the vulnerability and severity of common diseases such as type 2 diabetes, metabolic syndrome, cancer, kidney diseases, and obesity. Vitamin D adequacy is critical to overcoming infections and autoimmunity. Maintaining the population’s vitamin D sufficiency (above 40 ng/mL) and individuals above 50 ng/mL with vitamin D_3_ supplements and/or daily sun exposure is the most cost-effective way to reduce chronic diseases, sepsis, overcome viral epidemics, and autoimmune disorders, which provides better health and reduce healthcare costs. 

Maintaining serum 25(OH)D concentrations above 50 ng/mL improves overall health and reduces the severity of chronic diseases, infection and autoimmunity, and all-cause mortality. Furthermore, it minimizes infection-related complications, including COVID-19-related hospitalizations and deaths. Vitamin D sufficiency is the most cost-effective way to reduce illnesses, infections, and healthcare costs. It should be a part of routine public health and clinical care.

## 7. Conclusions

Maintenance of sufficient circulating 25(OH)D has a profound beneficial effect on the body. Such would decrease musculoskeletal disorders and many common extra-skeletal diseases and disorders, including insulin resistance, prediabetes, the severity of diabetes, metabolic syndrome, inflammation, autoimmunity, and so forth. In addition to its endocrine effects, vitamin D exerts genomic, membrane-based, and autocrine, and paracrine effects in peripheral target tissues subject to epigenesis modulation [288]. Maintaining mean population vitamin D status—serum 25(OH)D concentrations—above 40 ng/mL leads to broader benefits, better health, and reduced healthcare costs. Vitamin D sufficiency significantly impacts its physiological benefits, including reducing the risks of chronic diseases, infections, and all-cause mortality [289]. Instead of waiting for people to develop sicknesses and complications from chronic hypovitaminosis D-associated illnesses and treating them, maintaining the population's vitamin D sufficiency should be the way forward. This is the most cost-effective approach to keeping people healthy. Therefore, adopting this to clinical practice guidelines and healthcare insurance protocols is warranted.

## Figures and Tables

**Table 1:** Infections and autoimmune diseases significantly improved by vitamin D *.

Examples of Infections	Autoimmune Diseases and Others
Tuberculosis, leprosy, common cold (intracellular microorganisms)	Allergy/eczema
Influenza type A ^$^	Asthma
Coryza (common cold)	Chronic hives
Upper respiratory tract infections	Fibromyalgia
Lower urinary tract infections	Inflammatory bowel disease
Bacterial vaginosis in pregnant women	Multiple sclerosis
Periodontal gum disease and infections	Myositis and periostitis
Osteonecrosis of the jaw	Primary biliary cirrhosis
Miscellaneous fungal infections	Psoriasis
Yeast infection	Polyautoimmunity
Coxsackie A and B	Rheumatoid arthritis/ Behcet’s disease
SARS-CoV-2	Type 1 diabetes mellitus

* From multiple sources, including http://www.vitamindwiki.com/VitaminDWiki (accessed on 10 May 2023) [30,31,112]. ^$^ Influenza type B risk is not affected by vitamin D status [113].

## Data Availability

Not applicable.

## Abbreviations

1:25(OH)_2_D	1,25-dihydroxyvitamin D
25(OH)D	25-hydroxy vitamin D
BMI	Body mass index
CRP	C-reactive protein
CVD	Cardiovascular disease
D_3_	Cholecalciferol
PRISMA	Preferred Reporting Items for Systematic Reviews and Meta-Analyses
PCR	Polymerase chain reaction
PTH	Parathyroid hormone
RAS	Renin–angiotensin system
RCTs	Randomized controlled clinical trials
ROS	Reactive oxygen species
SR	Systematic Review
T1D	Type 1 diabetes mellitus
T2D	Type 2 diabetes mellitus
UVB	Ultraviolet B
VDR/CTR	Vitamin D (Calcitriol) receptor
VDBP	Vitamin D binding protein
VDR	Vitamin D receptor
